# Constitutive Activity among Orphan Class-A G Protein Coupled Receptors

**DOI:** 10.1371/journal.pone.0138463

**Published:** 2015-09-18

**Authors:** Adam L. Martin, Michael A. Steurer, Robert S. Aronstam

**Affiliations:** Department of Biological Sciences, Missouri University of Science and Technology, Rolla, MO, United States of America; Wake Forest University, UNITED STATES

## Abstract

The purpose of this study was to evaluate the extent of constitutive activity among orphan class-A G protein coupled receptors within the cAMP signaling pathway. Constitutive signaling was revealed by changes in gene expression under control of the cAMP response element. Gene expression was measured in Chinese hamster ovary cells transiently co-transfected with plasmids containing a luciferase reporter and orphan receptor. Criteria adopted for defining constitutive activation were: 1) 200% elevation over baseline reporter gene expression; 2) 40% inhibition of baseline expression; and 3) 40% inhibition of expression stimulated by 3 μM forskolin. Five patterns of activity were noted: 1) inhibition under both baseline and forskolin stimulated expression (GPR15, GPR17, GPR18, GPR20, GPR25, GPR27, GPR31, GPR32, GPR45, GPR57, GPR68, GPR83, GPR84, GPR132, GPR150, GPR176); 2) no effect on baseline expression, but inhibition of forskolin stimulated expression (GPR4, GPR26, GPR61, GPR62, GPR78, GPR101, GPR119); 3) elevation of baseline signaling coupled with inhibition of forskolin stimulated expression (GPR6, GPR12); 4) elevation of baseline signaling without inhibition of forskolin stimulated expression (GPR3, GPR21, GPR52, GPR65); and 5) no effect on expression (GPR1, GPR19, GPR22, GPR34, GPR35, GPR39, GPR63, GPR82, GPR85, GPR87). Constitutive activity was observed in 75% of the orphan class-A receptors examined (30 of 40). This constitutive signaling cannot be explained by simple overexpression of the receptor. Inhibition of cAMP mediated expression was far more common (65%) than stimulation of expression (15%). Orphan receptors that were closely related based on amino acid homology tended to have similar effects on gene expression. These results suggest that identification of inverse agonists may be a fruitful approach for categorizing these orphan receptors and targeting them for pharmacological intervention.

## Introduction

G protein coupled receptors (GPCR) comprise a large superfamily of receptors characterized by a seven transmembrane domain structure and an ability to activate intracellular transducer G proteins. Over 800 GPCRs in five main families have been identified in the human genome on the basis of sequence analysis [1]. These receptors can be activated by hormones, neurotransmitters, odorants, light, or pheromones. GPCRs for which the endogenous ligand is not known or is unclear are designated as orphan receptors. While little is known about many of these receptors, this family also includes receptors for which native activators have been tentatively identified, such as GPR3, GPR6, and GPR12, which are putative short chain fatty acid receptors [2], GPR65, which responds to pH changes [3], and GPR119, which has many potential agonists and has been the subject of multiple reviews [4],[5]. Detailed information on the status of individual orphan receptors can be found in the IUPHAR database [6]. The number of orphan G protein coupled receptors has decreased from 150 in 2004 [7] and to as few as 77 in 2014[8]. The first receptor to be “de-orphanized” (or “adopted”) was the 5-HT1A receptor [9]. This process continued with new methods that allowed high throughput screening for endogenous ligands for the remaining orphan receptors [10]. The rate of de-orphanization, however, appears to be slowing [11].

The phenomenon of ligand-independent “constitutive” signaling was first observed with the delta opioid receptor in 1989 [12]. This was followed by the discovery of mutant versions of other native GPCRs that signal in a similar manner [13]. Constitutive activity is now known to be present in a large number of GPCRs. As of this publication, a PubMed search for “GPCR” and “constitutive” reveals 132 references since 2010. Site directed mutagenesis has led to increased constitutive activity in all five muscarinic GPCR subtypes [14] and its application among orphans was reviewed in 1998 [15]. Structural analyses of some receptors suggest that this mutation eliminates interactions between hydrophobic amino acids on the third and sixth transmembrane, leading to the formation of a water filled pore [16] [17]. This led to a “unifying” theory on the biochemical mechanisms that regulate GPCR activation, including the changes that may lead to constitutive signaling [18].

Constitutive signaling as a tool in orphan receptor characterization was reviewed in 2006 [19]. The history of inverse-agonists (i.e., compounds that inhibit constitutive activity) as a therapeutic approach has also been reviewed [20]. While the use of constitutive signaling for drug discovery (notably, for inverse-agonists) has been discussed [10], the use of constitutive signaling to de-orphanize GPCRs has not been widely exploited.

The use of constitutive signaling poses certain challenges. There is the risk that endogenous ligands or activating conditions may be present in the testing media, thereby confounding data interpretation, as was the case for the ADORA2 receptor [21]. Receptors can also respond differently under different conditions, either due to promiscuous interactions with transducer elements, cellular conditions [22], or even hetero-dimerization with other native receptors [23]. Nevertheless, constitutive signaling has been useful in the discovery of native ligands [24], and is required for the systematic search for inverse agonists.

The prevalence of constitutive activity among Class-A orphan GPCRs has not been comprehensively examined. Accordingly, in the present study, we examined 40 Class- A orphan GPCRs to determine the prevalence of cAMP dependent constitutive signaling (i.e., signaling that is generally mediated by Gs and Gi transducer proteins), using receptor activation or inhibition of gene expression under control of the cAMP-dependent response element (CRE) as the indicator of pathway activation.

## Materials and Methods

Histamine, Muscarinic and Orphan GPCR receptor genes, cloned into pcDNA3.1+ (Life Technologies) were acquired from the MS&T cDNA Resource Center (www.cdna.org). These constructs were transiently co-transfected with Luciferase coupled reporter vectors to monitor CRE dependent gene expression. Each experimental treatment involved 4 wells seeded with 40,000 CHO-K1 cells in 96-well plates and incubated for 24 hours. Experiments were repeated 3 to 8 times. An “empty” plasmid (pcDNA3.1+) was used as a transfection negative control. Forskolin (3μM) mediated stimulation of adenylate-cyclase served as a positive control for the assay and additionally was used to normalize responses across experiments. Forskolin was administered 6 hours prior to measurements concurrently with sham dosing (media) where appropriate. Receptor activity was reflected by induction of luciferase expression under the control of the cAMP response element (CRE).

### Cell Culture

CHO-K1 wild-type Chinese hamster ovary cells were obtained directly from the American Type Culture Collection (ATCC, catalog# CCL-61) and maintained at 37°C with 5% CO_2_ as previously described [25]. Culture media consisted of 90% HyClone DMEM (without phenol red or additional L-glutamine) supplemented with 10% Hyclone FetalClone II (bovine serum product). Plates were allowed to reach 80% confluency before splitting or for use in subsequent experiments.

### Transfection

Approximately 40,000 CHO-K1 cells in 100 μl of media were plated onto a 96-well plate and allowed to attach overnight and incubated until they had reached 80% confluency. Firefly Luciferase reporter vector (pGL4.29, Promega) was mixed with pcDNA3.1+ plasmid DNA containing orphan receptor GPCR coding sequences (Missouri S&T cDNA Resource Center, www.cdna.org) or an empty pcDNA3.1+ vector (Life Technologies) at a final concentration of 250 ng each per well. Transfection was carried out following manufacturer’s suggestions (Lipofectamine 2000 Reagent, Life Technologies), followed by an 18 h incubation before use.

### Luciferase Assay

Transfection medium was removed and replaced with complete medium. Controls and treatments intended to evaluate inhibition of the cAMP pathway (i.e., putative Gα-i signaling) were treated with 3.0 μM Forskolin (Sigma Aldrich). The plate was then incubated for an additional 6 hours. Immediately prior to visualization, the medium within each well was replaced with 25 μl of DMEM (-phenol red) without serum.

Imaging was performed using a FLUOstar Omega (BMG Labtech) 96-well plate reader. Auto-injection of 25ul of Bright-Glo Luciferase Reagent (Promega) was followed by 2 minutes of rotary incubation. Relative Light Units (RLUs) were obtained for each well in series over 1 minute. Each 96-well plate consisted of 12 treatment groups with 4 replicates in each group. Each treatment group was separated by a row of unused wells to minimize light pollution.

### Data Analysis

Statistical analysis was performed in MiniTab, version 17, using a randomized complete block design. This variant of an ANOVA analysis takes differences between experiments (plates/blocks) into account and also allows for examination of treatment-block interaction. This statistical analysis allows for a strong isolation of treatment effect within the experiments.

Each experimental group (n = 4) was divided by the average of the positive control treatments (3 μM Forskolin stimulated cells with CRE-Luciferase and empty pcDNA3.1+, n = 4) to normalize results between plates. Each experimental treatment was then divided by its control (+/- 3 μM Forskolin) to determine the fractional stimulation or inhibition. Data was graphed as the average percent change over control between 4 to 8 plates with the over-all treatment p-value for each comparison indicated via either a single star for a threshold of 0.05, or a double star indicating a threshold of 0.01. Treatment effects that did not meet either of these thresholds were displayed individually and in red.

### Accession Numbers

Fasta sequences of all genes used in this study are available via the cDNA Resource Center (www.cdna.org). Open reading frames for gene constructs used in this study were matched to the following Genbank (http://www.ncbi.nlm.nih.gov/genbank/) accession numbers: GPR1 version 1 (NM_005279), GPR3 (NM_005281), GPR4 (NM_005282), GPR6 (NM_005284), GPR12 (NM_005288), GPR15 (NM_005290), GPR17 variant 3 (NM_001161416), GPR18 (NM_005292), GPR19 (NM_006143), GPR20 (NM_005293), GPR21 (NM_005294), GPR22 (NM_005295), GPR25 (NM_005298), GPR26 (NM_153442), GPR27 (NM_018971), GPR31 (NM_005299), GPR32 (NM_001506), GPR34 (NM_005300), GPR35 (AY275467), GPR39 (NM_001508), GPR45 (NM_007227), GPR52 (NM_005684), GPR55 (NM_005683), GPR57 variant 1 (AF_112461), GPR61 (NM_031936), GPR62 (NM_080865), GPR 63 variant 2 (NM_030784), GPR65 (NM_003608), GPR68 (NM_003485), GPR78 (NM_080819), GPR82 (NM_080817), GPR83 (NM_016540), GPR84 (NM_020370), GPR 85 (NM_018970), GPR87 (NM_023915), GPR101 (NM_054021), GPR119 (NM_178471), GPR132 (NM_013345), GPR150 (NM_199243), and GPR176(NM_007223), HRH4 (AY136745), hM2 (AF498916), hM3 (AF498917).

## Results and Discussion

Orphan receptors were judged to be constitutively active if they significantly affected cAMP dependent signaling (p < 0.05, according to a randomized complete block ANOVA, 4–8 experiments) and additionally fulfilled at least one of the following criteria: 1) 200% elevation over baseline reporter gene expression, 2) 40% inhibition of baseline expression, or 3) 40% inhibition of expression stimulated by 3 μM forskolin. These criteria were chosen to reflect thresholds large enough to minimize false-positives due to receptor over-expression. Among the 40 orphan receptors evaluated, 75% (30) met criteria for constitutive activity.

GPCR’s are characterized by their interaction with specific transducer G proteins. Gα-s and Gα-i play opposing roles in modulating cAMP levels in response to external stimuli by mediating the activation and inhibition of adenylate cyclase, respectively. However, crosstalk between signaling pathways has been demonstrated and can take place at multiple levels. For example, signaling potential can be affected by the level of receptor expression [26], receptor interaction with different transducer proteins [27] and second messenger (ie. calcium, cAMP) stimulation of kinases can affect (stimulate or inhibit) other pathways. Accordingly, present results on cAMP metabolism cannot be unambiguously ascribed to specific signaling pathways (i.e. Gα-s or Gα-i). Additional confounding factors related to the nature of the experiment are possible (e.g. long term exposure to Forskolin, and the potential impacts of constitutive activity itself).

In the present experiments five patterns of signaling were noted.

### Group A: Receptors displaying constitutive inhibition of both baseline (B) and forskolin stimulated (F) CRE-mediated gene expression

**Fig 1:**
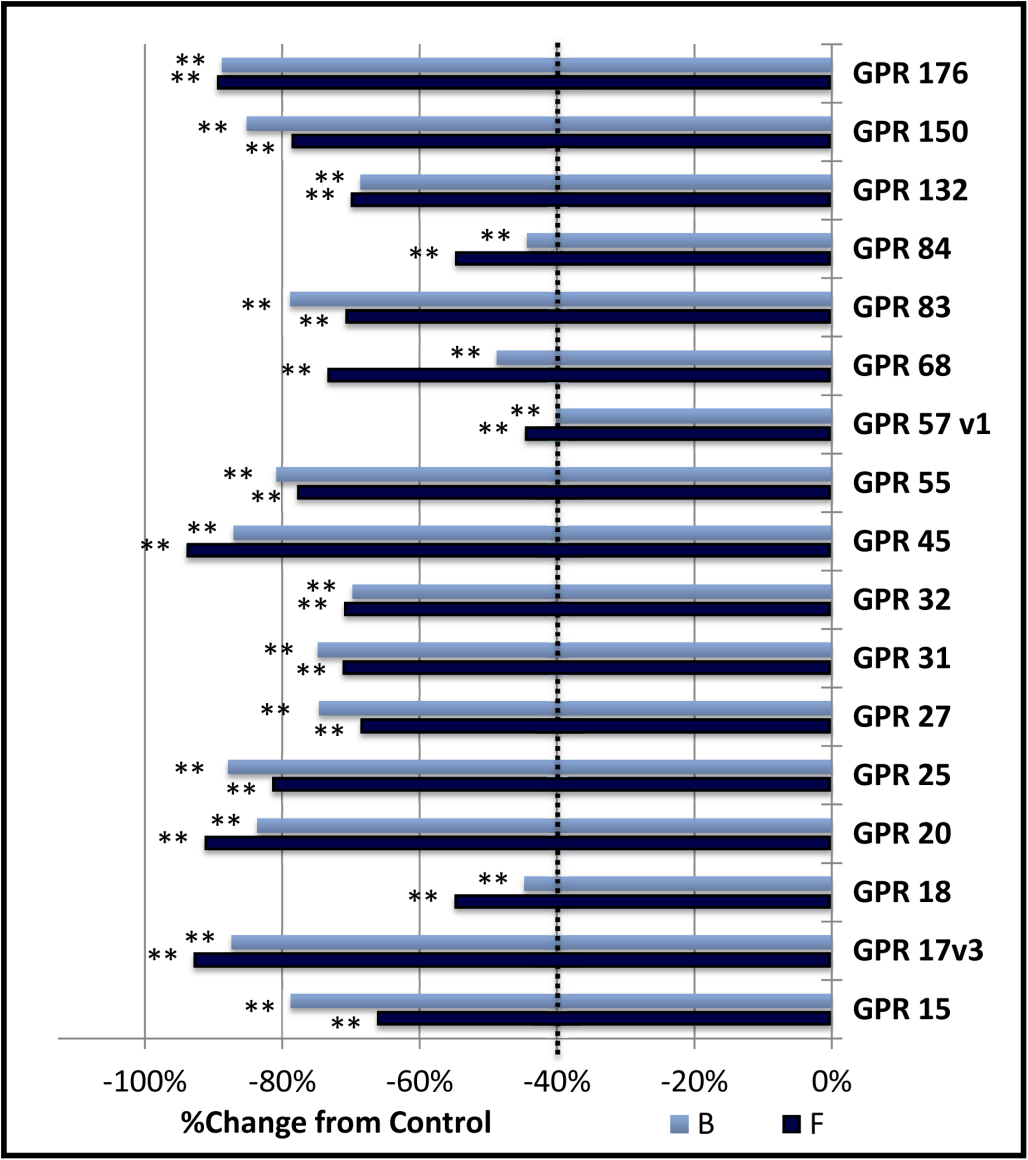
Group A: Constitutive inhibition of both baseline (B) and forskolin stimulated expression (F).

This largest group of receptors (17 of 40, Fig 1) exhibited significant constitutive inhibition of CRE-mediated gene expression under both baseline and forskolin-stimulated conditions. This group is comprised of GPR15, GPR17 variant 3, GPR18, GPR20, GPR25, GPR27, GPR31, GPR32, GPR45, GPR55, GPR57 variant 1, GPR68, GPR83, GPR84, GPR132, GPR150, and GPR176. In all cases, the statistical significance level was less than 0.01.

The percent change of cAMP-dependent signaling in CHO-K1 cells transfected with CRE-Luciferase reporter vector and an orphan G protein coupled receptors is shown. Activity was measured as relative light units (RLU) and normalized between experiments by dividing by the average of the 3 μM Forskolin stimulated control within each plate. This value was then divided by the control for each condition to obtain the fractional change, with a value of zero indicating no change from control levels. Vertical dotted lines represent the minimum signaling threshold to be scored as constitutively active within this study. Changes in basal cAMP dependent expression are indicated by the light blue bars labeled “B”. Changes in expression in the presence of 3 μM Forskolin (6 hour exposure) is indicated by the dark blue bars labeled “F”. All receptors presented in this figure showed a significant treatment effect (** = p < .01, standard error not shown) and met the criteria adopted in this study to define constitutive activity.

This behavior is similar to results we obtained during preliminary experiments with the histamine receptor 4 (HRH4), a receptor shown to signal constitutively through the Gα-i pathway in other labs [28]. While all 17 of these receptors inhibited gene expression by over 40%, five of them inhibited cAMP dependent gene expression by over 80%.

### Group B: Receptors displaying constitutive stimulation of baseline (B) and constitutive inhibition of forskolin stimulated CRE-mediated gene expression (F)

**Fig 2:**
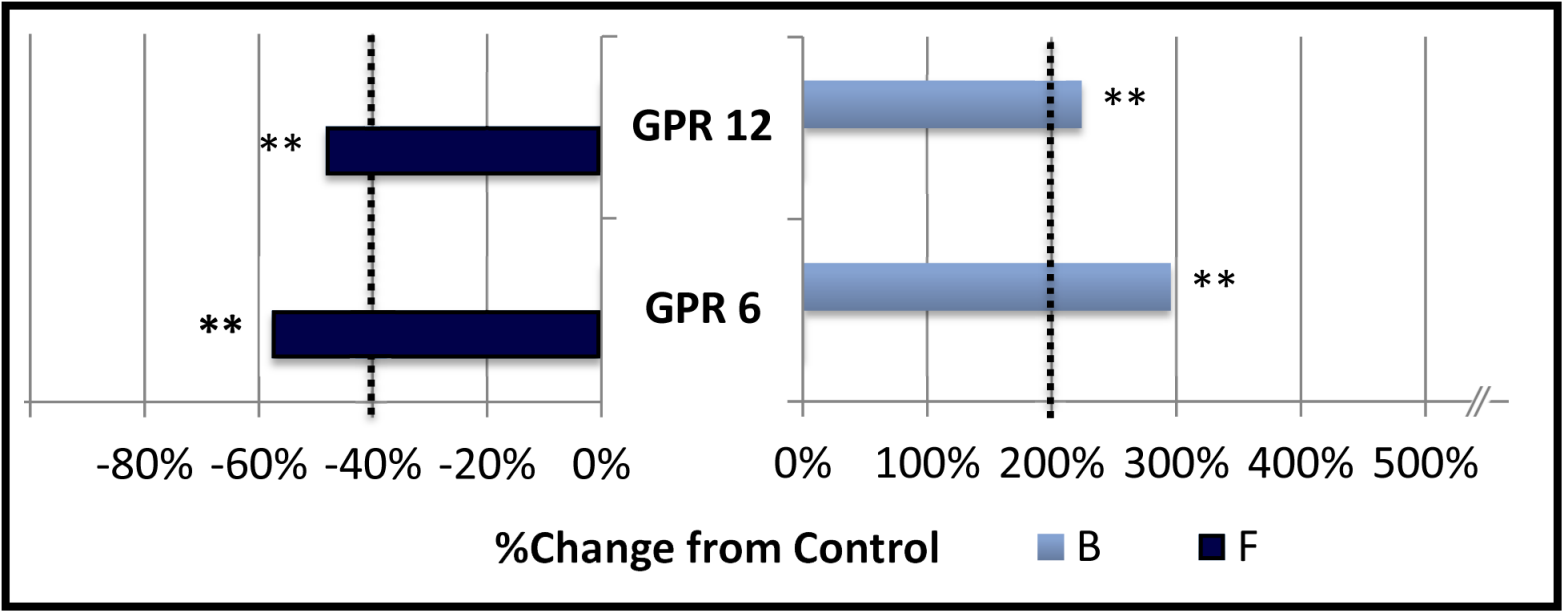
Group B: Constitutive stimulation of baseline (B) and constitutive inhibition of forskolin stimulated expression (F).

This group (Fig 2) is comprised of receptors that are closely related in terms of amino acid homology: GPR6 and GPR12 [29]. A third member of this family (GPR3) produced extremely variable effects on forskolin stimulated expression and its inhibition within these experiments was not statistically significant (p = 0.072). Accordingly, it was included in group D.

The percent change in cAMP dependent signaling in CHO-K1 cells transfected with CRE-Luciferase reporter vector and various orphan G protein coupled receptors is shown. Vertical dotted lines represent the minimum signaling threshold to be scored as constitutively active within this study. Activity was measured, normalized and graphed as described in the legend to Fig 1. Changes in basal cAMP dependent expression are indicated by the light blue bars labeled “B”. Changes in expression in the presence of 3 μM Forskolin (6 hour exposure) is indicated by the dark blue bars labeled “F”. Members of this group showed a significant treatment effect (** = p < .01, standard error not shown) and met the criteria adopted in this study to define constitutive activity.

Receptors in this group exhibited constitutive stimulation of CRE-mediated gene expression under baseline conditions while inhibiting CRE-mediated gene expression stimulated by 3 μM forskolin. Thus, these GPCRs can constitutively stimulate at least one aspect of baseline cAMP-mediated signaling (i.e., CRE mediated gene expression) while inhibiting high levels of cAMP-mediated signaling induced by an exogenous agent (forskolin). It is possible that these receptors act to maintain an elevated but controlled homeostatic level of cAMP by this pathway, a function known to be present in maintenance of meiotic arrest in oocyte development [30]. While this work does not measure activation of specific pathways, this behavior could be explained by preferential activation of a stimulatory pathway under low cAMP levels (possibly Gs) and a switch to inhibitory pathways at elevated levels of cAMP (possibly Gi). Further analysis is required to discover the underlying mechanisms that lead to this response.

### Group C: Receptors with no effect on baseline expression (B) but inhibit forskolin stimulated gene expression (F)

**Fig 3:**
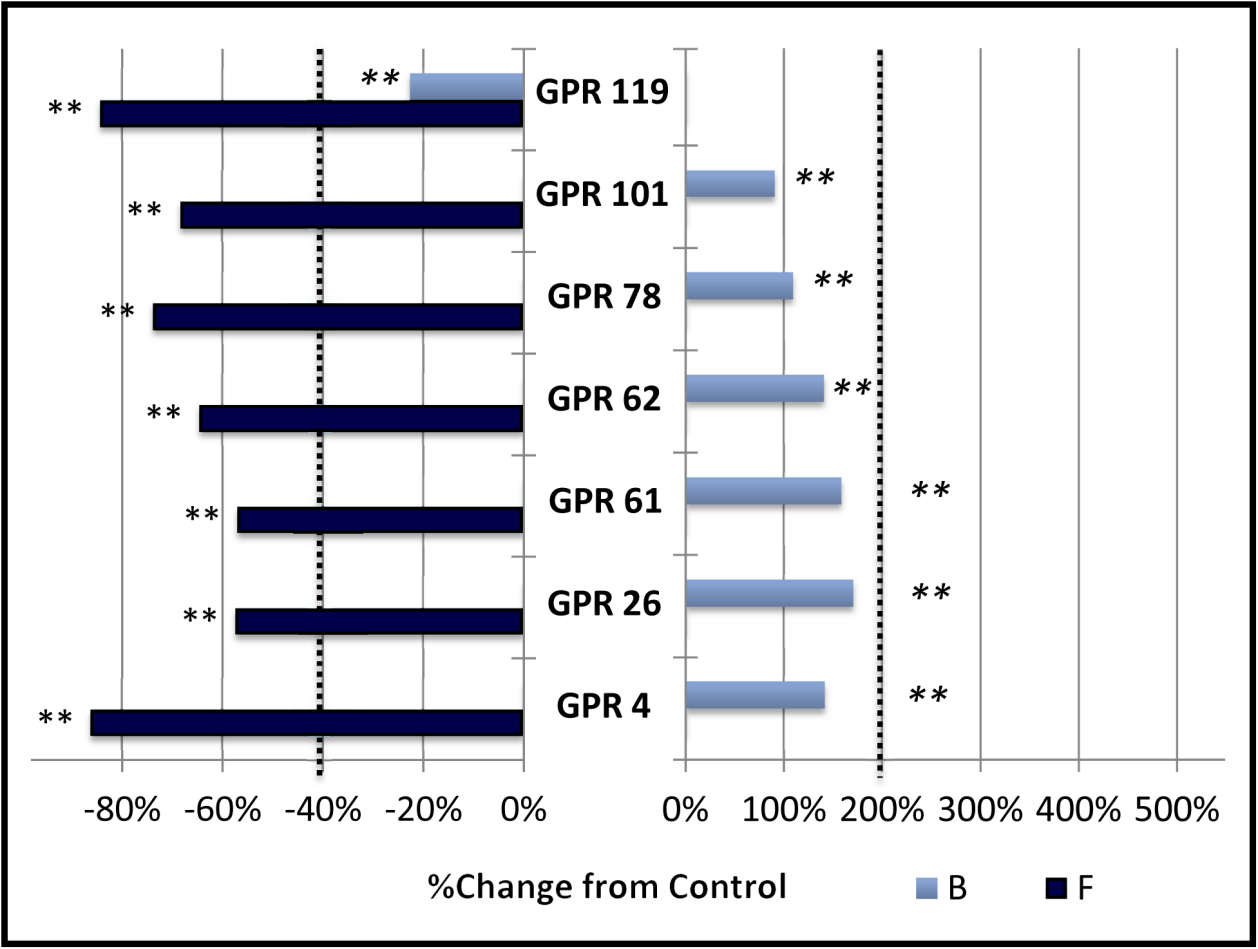
Group C: No effect on baseline (B) and constitutive inhibition of forskolin stimulated expression (F).

This group (Fig 3) is comprised of GPR4, GPR26, GPR61, GPR62, GPR78, GPR101, and GPR119. These receptors did not alter baseline signaling enough to meet our criteria for constitutive activity, although they all inhibited CRE mediated gene expression stimulated by 3 μM forskolin by at least 40%. In this way, they are similar to results we obtained with a constitutively active mutant version of the M2 acetylcholine receptor[31], which others have shown is capable of signaling through both the Gα-s and Gα-i pathways [32,33].

The percent change in cAMP dependent signaling in CHO-K1 cells transfected with CRE-Luciferase reporter vector and various orphan G protein coupled receptors is shown. Activity was measured, normalized and graphed as described in the legend to Fig 1. Vertical dotted lines represent the minimum signaling threshold to be scored as constitutively active within this study. Changes in basal cAMP dependent expression are indicated by the light blue bars labeled “B”. Changes in expression in the presence of 3 μM Forskolin (6 hour exposure) is indicated by the dark blue bars labeled “F”. Members of this group showed significant treatment effect (** = p < .01, standard error not shown) but did not meet criteria for constitutive activation of 200% stimulation over baseline expression levels (B). All members displayed constitutive inhibition (40% or more) of 3 μM forskolin stimulated expression (F).

While baseline stimulation did not meet criteria for constitutive activity as defined in this study, many of the receptors in this group produced a very significant “block” and “treatment by block” effect (p < 0.01). Further measurements may reveal constitutive activation of cAMP signaling under other conditions.

### Group D: Receptors displaying stimulation of baseline (B) but no inhibition of forskolin stimulated expression (F)

**Fig 4:**
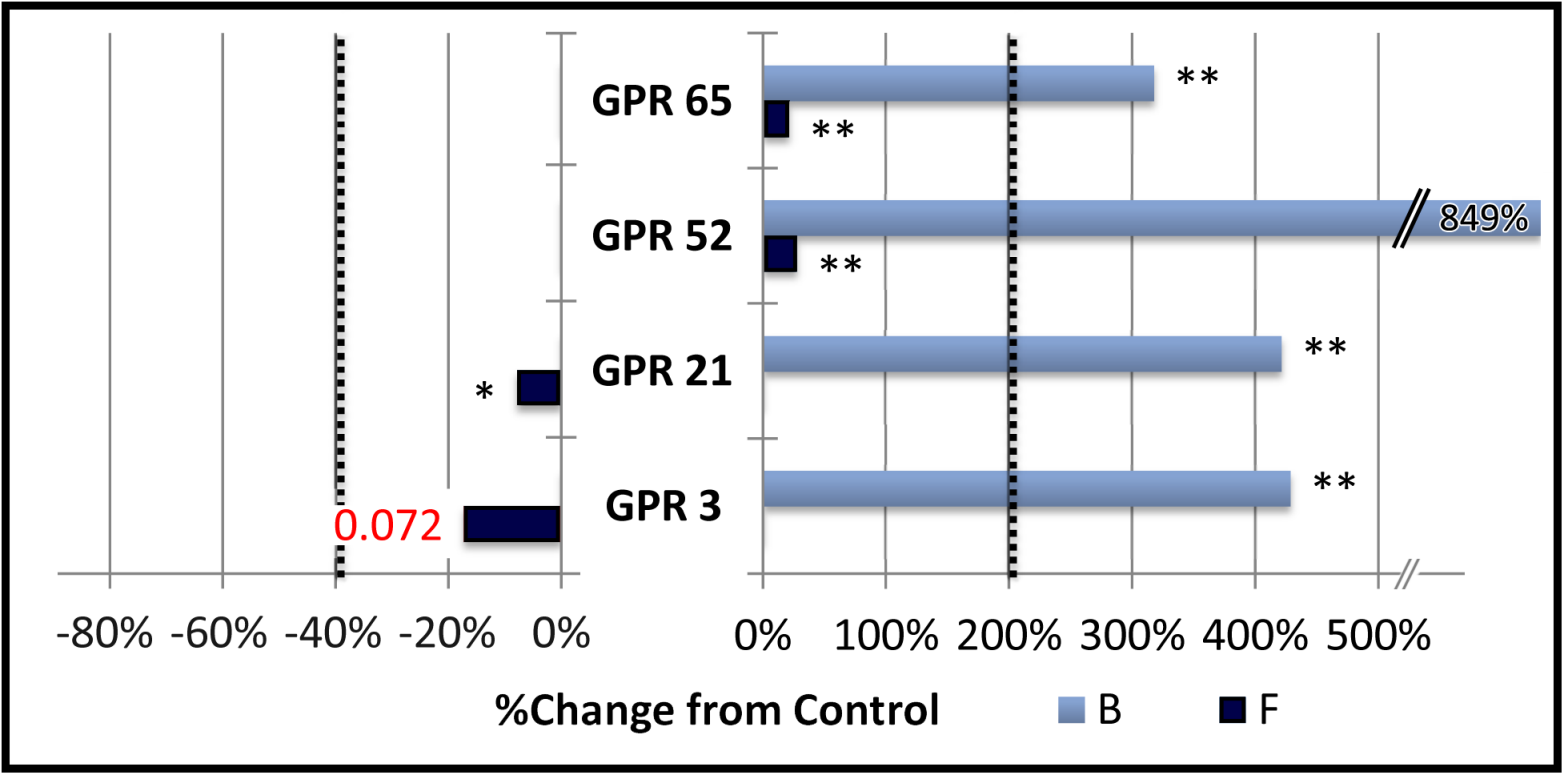
Group D: Stimulation of baseline (B) but no inhibition of forskolin stimulated expression (F).

This group (Fig 4) was comprised of GPR3 and GPR65 along with the closely related GPR21 and GPR52 [29]. These receptors exhibited constitutive stimulation of baseline cAMP dependent signaling without any constitutive inhibition of the signaling stimulated by 3 μM forskolin. This is similar to CRE-mediated responses we have noted with a constitutively active mutant of the M3 human muscarinic acetylcholine receptor which raises baseline and potentiates forskolin response by signaling through Gα-q and Gα-s pathways [31].

The percent change in cAMP dependent signaling in CHO-K1 cells transfected with CRE-Luciferase reporter vector and various orphan G protein coupled receptors is shown. Activity was measured, normalized and graphed as described in the legend to Fig 1. Vertical dotted lines represent the minimum signaling threshold to be scored as constitutively active within this study. Changes in basal cAMP dependent expression are indicated by the light blue bars labeled “B”. Changes in expression in the presence of 3 μM Forskolin (6 hour exposure) is indicated by the dark blue bars labeled “F”. Members of this group showed a significant treatment effect (* = p < .05, ** = p < .01, standard error not shown) and an increase of CRE-mediated gene expression of more than 200% under baseline conditions (B), but did not affect gene expression stimulated by 3 μM forskolin (F).

### Group E: Receptors that do not display constitutive activity

**Fig 5:**
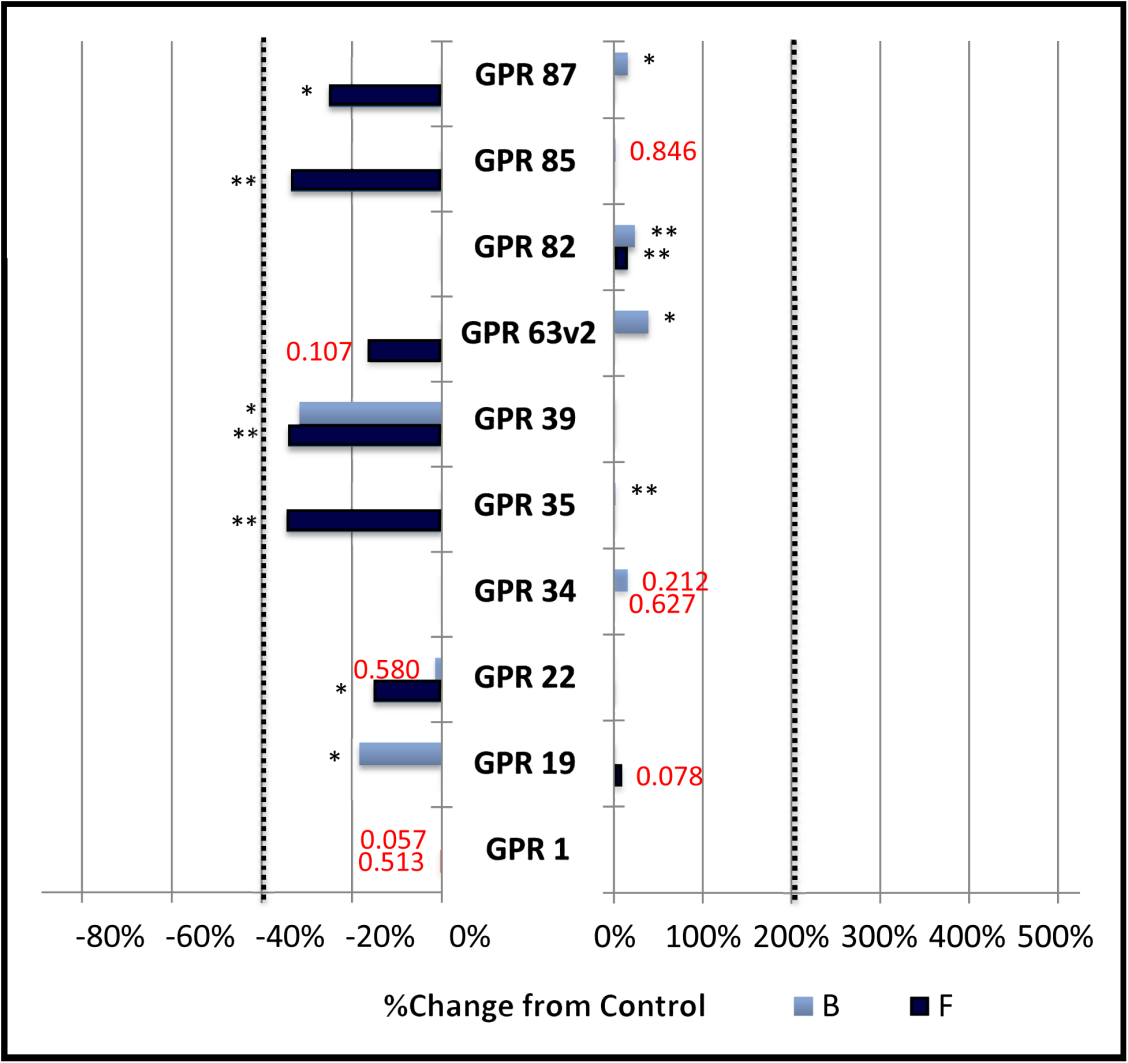
Group E: No constitutive activity.

This group (Fig 5) is comprised of the remaining 10 orphan receptors: GPR1, GPR19, GPR22, GPR34, GPR35, GPR39, GPR63 variant 2, GPR82, GPR85, and GPR87.These receptors lacked constitutive activity insofar as they failed to either have a significant treatment effect or meet at least one of the three criteria for constitutive activity (i.e., 200% baseline stimulation, 40% inhibition of baseline, or 40% inhibition of forskolin-stimulated activity). Thus, not all orphan receptors exhibit constitutive signaling by our criteria. Accordingly, the constitutive activity noted in the present study is unlikely to be due to an artifact arising solely from overexpression of receptor proteins in this system. While surface expression of individual orphan receptors was not assayed in this study and cannot be completely ruled out, it is likely not a primary factor as all orphans assayed here-in were cloned into the same expression plasmid and therefore under the control of the same (CMV) promotor.

The percent change in cAMP dependent signaling in CHO-K1 cells transfected with CRE-Luciferase reporter vector and various orphan G protein coupled receptors. Activity was measured, normalized and graphed as described in the legend to Fig 1. Vertical dotted lines represent the minimum signaling threshold to be scored as constitutively active within this study. Changes in basal cAMP dependent expression are indicated by the light blue bars labeled “B”. Changes in expression in the presence of 3 μM Forskolin (6 hour exposure) is indicated by the dark blue bars labeled “F”. Receptors in this group failed to meet either study criteria of a significant treatment effect (p value listed in red, standard error not shown) and/or threshold for constitutive signaling.

Several of the receptors in this group displayed large fluctuations in response from plate to plate, resulting in either loss of a significant treatment affect, or very significant “block” and/or “treatment by block” effect (p < .01). The reasons for this variability are not understood but suggest the presence of undefined variables in these multistep pathways. Transfection efficiency is not a likely explanation for this variability due to the roughly five to one ration of Forskolin to baseline controls maintained throughout experimental plates. It is also possible that orphans included in this group, or the previous ones, may yet reveal constitutive signaling via non cAMP dependent mechanisms in subsequent studies, further increasing the potential power of this signaling mechanism.

## Conclusions

The purpose of this study was to identify the prevalence of constitutive activity in the cAMP-dependent signaling pathway within 40 Class-A orphan GPCRs using a luciferase-linked gene expression system. The activities examined were 1) stimulation of baseline signaling, 2) inhibition of baseline signaling, and 3) inhibition of forskolin-stimulated signaling. While 10 of the 40 receptors examined did not display constitutive activity, cAMP-dependent constitutive activity was observed in 75% of the orphan class-A receptors transiently expressed in CHO-K1 cells. Five groups of receptors were defined reflecting different effects on baseline and forskolin-stimulated expression. Constitutive inhibition of cAMP-dependent signaling was much more common than stimulation (26 vs. 6 receptors). Receptors that are closely related on the basis of amino acid homology displayed similar response patterns. For instance, the closely related GPR3, GPR6, and GPR12 all stimulated baseline cAMP-dependent signaling while GPR6 and GPR12 both inhibited forskolin activated signaling. Similarly, receptors in a second closely related group (GPR21 and GPR52) both stimulated cAMP-dependent signaling without inhibiting activity in the presence of forskolin. These results indicate that constitutive signaling is an important physiological property of most of the remaining orphan class-A GPCRs and may be a reason that many of their native ligands remain elusive. This suggests that a search for inverse agonists may be the most effective approach to understanding their physiological roles as well as selecting targets for pharmacological intervention.
